# C-reactive protein is not a screening tool for late periprosthetic joint infection

**DOI:** 10.1186/s10195-020-0542-2

**Published:** 2020-02-24

**Authors:** Bernd Fink, Michael Schlumberger, Julian Beyersdorff, Philipp Schuster

**Affiliations:** 1 Department for Joint Replacement, Rheumatoid and General Orthopaedics, Orthopaedic Clinic Markgröningen, Kurt-Lindemann-Weg 10, 71706 Markgröningen, Germany; 2 Orthopaedic Department, University Hospital Hamburg-Eppendorf, Hamburg, Germany; 3 Department of Orthopedics and Traumatology, Clinic Nuremberg,, Paracelsus Medical Private University, Nuremberg, Germany

**Keywords:** Periprosthetic joint infection, Diagnostic, C-reactive protein

## Abstract

**Background:**

Preoperative diagnosis of periprosthetic joint infection (PJI) is important because of the therapeutic consequences. The aim of the present study is to investigate whether the serum C-reactive protein (CRP) level can be used as a screening tool for late PJI.

**Materials and methods:**

A cohort of 390 patients with revision surgery of total hip prostheses (200) or total knee prostheses (190) was assessed for late PJI by determining CRP serum level and performing preoperative aspiration with cultivation and intraoperative tissue analyses with cultivation and histologic examination, using the Musculoskeletal Infection Society (MSIS) and International Consensus Meeting (ICM) criteria.

**Results:**

A total of 180 joints were rated as PJI (prevalence 46%). Of these, 42.8% (77) showed a CRP level below 10 mg/L and 28.3% (51) showed a normal CRP level of less than 5 mg/L. The 76.9% of the cases with slow-growing bacteria showed a CRP level below 10 mg/L, and 61.5% showed a normal CRP level.

**Conclusions:**

Serum CRP level should not be used as a screening tool to rule out late PJI.

**Level of evidence:**

Level 2 (diagnostic study).

## Introduction

Periprosthetic joint infection (PJI) is a devastating complication of arthroplasty procedures and has many consequences. The level of incidence for total hip and knee arthroplasties ranges between 1% and 2%, on average [1]. However, in some reports, this type of infection is claimed to be the most frequent cause of implant failure during the first 5 years following implantation [2, 3]. Thus the accuracy of the preoperative diagnosis of possible periprosthetic joint infection becomes important, especially in cases of loosened or painful endoprostheses [4–6].

Whereas early infections, i.e. those occurring within the first 4 weeks of implantation, usually cause local and systemic inflammatory reactions, these parameters are often missing in cases of late PJI. Even though a new definition for late periprosthetic joint infection (PJI) with a high sensitivity of 97.7% has been introduced, the diagnosis of late PJI before revision surgery remains difficult because often only a small amount of synovial fluid is aspirated, which cannot be used for all diagnostic tests described in this definition [7]. Moreover, some of the diagnostic tests are related to intraoperative findings. As a result, many surgeons use CRP as a first-line screening test for late PJI, with a threshold of mostly between 10 and 20 mg/L, and only perform further diagnostic tests in patients with abnormal CRP levels [6, 8–11]. However, other studies have shown that the CRP is associated with a significant amount of false-negative results—between 11% and 35%—such that CRP could lead to a misdiagnosis of arthroplasties affected by PJI [12–16].

Therefore, the objective of the present study is to investigate whether serum CRP level is an adequate screening tool to rule out late PJI, as still used by several surgeons [7, 8]. Additionally, the following questions should be answered:

- What percentage of PJI presents CRP levels below 10 mg/L?
- What percentage of PJI presents normal CRP levels (< 5 mg/L)?
- Are there differences for the CRP level in cases of fast- and slow-growing bacteria [17]?

## Materials and methods

This retrospective analysis of a prospective collected database included 408 patients who underwent revision surgery for loosening of total hip (THA) or knee arthroplasties (TKA) between January 2016 and December 2018. Prostheses with acute periprosthetic joint infection were not included. Moreover, systemic inflammatory diseases such as rheumatoid arthritis were excluded because of the possible influence on serum CRP levels, leaving 390 patients (191 men, 199 woman) aged 68 ± 11 (29–87) years who underwent revision surgery for loosening of THA (190) and TKA (200). Revision surgery was carried out 38.6 ± 38.1 months (3–210 months) after primary implantation. All cases underwent a prior aspiration of the joint. None of the patients took any antibiotics within the 4 weeks preceding the aspiration. Joint aspiration was carried out under aseptic conditions. The harvested fluid was immediately transferred into paediatric blood culture bottles containing BD BACTEC-PEDS-PLUS/F-Medium (Becton Dickinson; Heidelberg, Germany) and incubated for 14 days [17].

During the revision surgery itself, samples were taken from five different areas close to the prosthesis (synovium and periprosthetic connective tissue membrane). In addition, five samples from the synovium and the periprosthetic connective tissue membrane associated with the loosened prosthesis were obtained for histological assessment. Perioperative antibiotics were administered once all the samples had been taken. The biopsy samples were each placed in sterile tubes and transferred to the microbiological laboratory within 1 h of sampling, as was done for the aspirated fluid. The samples were streaked onto blood agar and inoculated into special nutrient broth for anaerobic organisms. All the samples were incubated for 14 days [16]. The results were analysed according to Virolainen et al. [18], Pandey et al. [19], the criteria of the Musculoskeletal Infection Society (MSIS) [10] and the 2018 criteria for late PJI of the International Consensus Meeting (ICM) [7], whereby a prosthesis is regarded as infected when at least one of the following conditions is fulfilled:

1. Demonstration of the same pathogen in at least two of the samples (tissue and/or aspiration fluid)
2. Demonstration of a pathogen in at least one sample and demonstration of at least five neutrophilic polymorph leukocytes in five high-power field (×400) in the associated histological preparation

The presence of bacteria in only one sample without any histological confirmation was regarded as a result of contamination during the sampling procedure or during the incubation period, in accordance with Virolainen et al. [18].

The diagnosis obtained from the revision surgery samples together with the aspiration was regarded as the definitive result with respect to periprosthetic infection and was then used to evaluate the diagnostic method based on serum CRP level.

The conditional probabilities “sensitivity” and “specificity” were determined as characteristic parameters of the diagnostic methods. In the case of sensitivity, this represents the proportion of infections that the test detects as infected (the positive test results) and, in the case of specificity, the proportion of tests that have negative results. The probability that, in cases of positive or negative test results, infection does or does not exist is represented by the positive and negative prediction percentages. They are dependent on the prevalence, i.e. the proportion of infected prostheses in the whole collective, or in other words, the pre-test probability of infection. Bayes equation was used to calculate these statistics [20]. Statistical evaluation was performed using SPSS for Windows (version 22, IBM Corp.; Armonk, NY). The chi-square test was used for comparison of nominal variables between groups. For statistical evaluation of nonparametric data, the Mann–Whitney *U*-test was used for unrelated samples, and the Wilcoxon signed-rank test was used for related samples. Student’s *t*-test was used for parametric data. For threshold calculation of the CRP, a receiver operating characteristics curve (ROC-curve) was used. The level of significance was set at *p* < 0.05. All subjects gave informed consent to participate in the study, and the protocol was approved by the research ethic board of the respective institution.

## Results

Of the 390 revision operations carried out, 180 were classified as infected, according to the criteria described above (prevalence = 46%). There were 82 total knee arthroplasties and 98 total hip arthroplasties infected in the 180 patients [128 men, 52 women; aged 68 ± 10 (31–87) years] The bacteria that were identified are listed in Table 1, whereby it should be noted that fast-growing bacteria (*Staphylococcus aureus*, coagulase-neg. staphylococci, *Enterococcus* species, *Streptococcus* species, and *Enterobacteriaceae*) were identified in 112 cases (62.2%), slow growing-bacteria (coryneform bacteria, *Cutibacterium* species, *Peptostreptococcus* species, and others with a growing period of more than 7 days on average) in 26 cases (14.4%) and two or more microorganisms from both groups in 42 cases (23.3%) [16]. Patients with fast-growing bacteria showed a significant higher CRP level (52.3 ± 67.8 mg/L) than patients with slow-growing bacteria (CRP 17.3 ± 39.4 mg/L, *p* < 0.001).

**Table 1:** Number of detected species

Group of bacteria	Bacteria	Numbers
Fast-growing bacteria	*Staphylococcus aureus*	25
	Coagulase-negative staphylococci	148
	*Enterococcus* species	3
	*Streptococcus* species	13
	*Enterobacteriaceae*	8
Slow-growing bacteria	Coryneform bacteria	14
	*Cutibacterium* species	52
	*Peptostreptococcus* species	3
	Other	15

In 109 cases, one species was found, and in 71 cases, two or more species were detected. The distribution of fast- and slow-growing bacteria is according to Schäfer et al. [13]

**Fig. 1:**
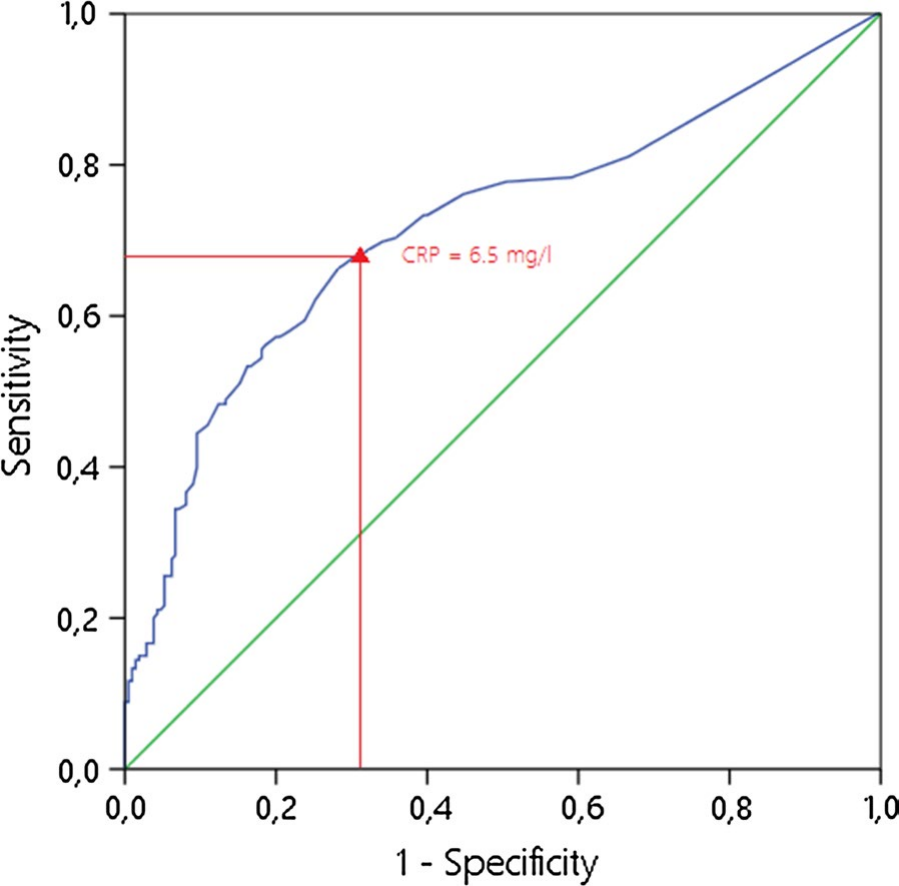
Receiver operating characteristics (ROC) curve for C-reactive protein (CRP) level in the serum. The best possible threshold is the point on the blue curve that is closest to the upper-left corner. In this case, the area under the curve (AUC) is 0.717, which means that there is a fair level of accuracy. However, the optimal threshold in this case is a CRP level of 6.5 mg/L, giving a sensitivity of only 68.3% and a specificity of only 66.6%

Seventy-seven (42.8%) of the 180 patients with PJI showed a CRP level below 10 mg/L. Hereby, 28.6% of the patients with fast-growing bacteria and 76.9% with slow-growing bacteria showed a CRP level below 10 mg/L (*p* < 0.001). A total of 51 patients (28.3%) with infections showed a normal CRP level below 5 mg/L. Of the infections with fast-growing bacteria, 17% showed a CRP level below 5 mg/L, and of those with slow-growing bacteria, 61.5% showed a normal CRP level (*p* < 0.001). With the cut-off of 10 mg/L for the CRP, a sensitivity of 57.2%, a specificity of 79.0%, a positive predictive value of 70.1% and a negative predictive value of 68.3% were calculated. Changing the cut-off to the level of a normal CRP at 5 mg/L leads to a sensitivity of 71.7%, a specificity of 62.4%, a positive predictive value of 62.0% and a negative predictive value of 72.0%. The ROC curve model revealed a CRP level of 6.5 mg/L as the best available threshold. However, at this particular threshold, sensitivity and specificity were calculated to be 68.3% and 66.6%, respectively, emphasising the very low quality of this diagnostic test for detection of PJI (Fig. 1). Analysis of the data shows that a cut-off level for the CRP, where sufficient sensitivities or specificities of around 90% could be reached, could not actually be calculated .

## Discussion

A preoperative examination to rule out PJI should be carried out before a loosened or painful prosthesis is exchanged because the presence of PJI would result in significant changes in the nature of the subsequent therapeutic procedures. Therefore, many authors recommend using serum CRP analysis (with a cut-off value of between 10 and 20 mg/L) as a screening tool for late PJI, with this analysis to be carried out before a revision operation takes place [6, 8–11]. Consequently, they only perform further examinations, such as joint aspiration, when the CRP level is elevated. This study clearly shows that this procedure cannot be performed to rule out PJI with high accuracy because 42% of infected prostheses would be misdiagnosed as aseptic loosening. Even changing the cut-off value to the normal level of CRP at 5 mg/L does not result in a sufficient screening test, because 28% of the PJIs would still not be detected. Especially late PJIs with slow-growing bacteria would not be detected when using CRP as a screening tool, because 77% of the patients in this study with PJI showed a CRP level below 10 mg/L, and 61% showed a normal CRP level below 5 mg/L.

The results of this study are in accordance with those of Pérez-Prieto et al. [14] and Akgün et al. [15], who found a CRP level below 10 mg/L in around one-third of their PJIs. The reason why we found an even higher percentage of PJIs with CRP level under 10 mg/L may be that, in the study of Akgün et al. [15], patients with acute PJI were also included.

The strength of our study is the homogeneity of the patient group, with a high prevalence of PJI. Our clinic is a reference centre for PJI treatment, which may explain this high prevalence. Patients with acute infections and systemic inflammatory diseases (such as rheumatoid arthritis) were excluded, as were those with an antibiotic treatment during the 4 weeks prior to diagnosis. The reason for this is that these diseases and treatment influence the serum CRP level [13]. However, the necessity to exclude patients with systemic inflammatory diseases and antibiotic treatment further reduces the validity of the serum CRP as a screening tool for PJI. Similarly, the inability to calculate a cut-off level for the CRP where sufficient sensitivities or specificities of around 90% could be reached emphasises the unreliability of this proposed diagnostic tool.

This study also has its limitations. We cannot completely rule out that some of the infected cases were misdiagnosed. The method used in this study was shown to possess a high accuracy of 98% and 93% in other studies [13]. However, adjusting for these individual cases would not improve the validity of the serum CRP significantly. Another limitation may be the fact that 42 cases (23.3%) could not be clearly assigned to infections with fast- or slow-growing bacteria because they had infections derived from two or more bacteria from both groups. However, the results of the reduced number of cases were still significant between the fast- and slow-growing group.

In summary, this study clearly shows that the serum CRP level should not be used as a screening tool to rule out late PJI. Therefore we recommend performing a joint aspiration before revision surgery of a loosened or painful joint prosthesis. Furthermore, we recommend using a combination of different tests such as cultivation, alpha-defensin or cell count, because no single diagnostic analysis has an accuracy of 100% [21, 22].

## Data Availability

The datasets used and/or analysed during the current study are available from the corresponding author on reasonable request.

## Abbreviations

CRP: C-reactive protein
ICM: International Consensus Meeting
mg/L: milligram per litre
MSIS: Musculoskeletal Infection Society
PJI: periprosthetic joint infection
ROC curve: receiver operating characteristic curve
